# Investigating the effects of two novel 4-MMPB analogs as potent lipoxygenase inhibitors for prostate cancer treatment

**DOI:** 10.1186/s40709-021-00141-w

**Published:** 2021-05-04

**Authors:** Sonia Iranpour, Aseel Kamil Mohammed Al-Mosawi, Ahmad Reza Bahrami, Hamid Sadeghian, Maryam M. Matin

**Affiliations:** 1 Department of Biology, Faculty of Science, Ferdowsi University of Mashhad, Mashhad, Iran; 2 Department of Biology, Faculty of Science, University of Thi-Qar, Nasiriyah, Iraq; 3 Industrial Biotechnology Research Group, Institute of Biotechnology, Ferdowsi University of Mashhad, Mashhad, Iran; 4 Applied Biomedical Research Center, Department of Laboratory Sciences, School of Paramedical Sciences, Mashhad University of Medical Sciences, Mashhad, Iran; 5 Novel Diagnostics and Therapeutics Research Group, Institute of Biotechnology, Ferdowsi University of Mashhad, Mashhad, Iran

**Keywords:** Prostate cancer, Lipoxygenases, Chemotherapy, Targeted therapy

## Abstract

**Background:**

Lipoxygenases are one of the critical signaling mediators which can be targeted for human prostate cancer (PC) therapy. In this study, 4-methyl-2-(4-methylpiperazinyl)pyrimido[4,5-b]benzothiazine (4-MMPB) and its two analogs, 4-propyl-2-(4-methylpiperazinyl)pyrimido[4,5-b]benzothiazine (4-PMPB) and 4-ethyl-2-(4-methylpiperazinyl)pyrimido[4,5-b]benzothiazine (4-EMPB), were proposed to have anti-tumor properties in prostate cancer.

**Methods:**

After synthesizing the compounds, cytotoxic effects of 4-MMPB and its two analogs against PC-3 cancerous and HDF normal cells were investigated by 3-[4,5-dimethylthiazol-2-yl]-2,5-diphenyltetrazolium bromide (MTT) assay and then mechanism of cell death was assessed by flow cytometry. Finally, the anti-tumor effects of the mentioned compounds were investigated in an immunocompromised C57BL/6 mouse model.

**Results:**

4-PMPB and 4-EMPB had similar anti-cancer effects on PC-3 cells as compared with 4-MMPB, while they were not effective on normal cells. Moreover, apoptosis and ferroptosis were the main mechanisms of induced cell death in these cancerous cells. Furthermore, in vivo results indicated that both analogs had similar anti-cancer effects as 4-MMPB, leading to delayed tumor growth without any noticeable side effects in weight loss and histological investigations.

**Conclusion:**

Thus, our results suggest that specific targeting of lipoxygenases via 4-MMPB analogs can be considered as a treatment of choice for PC therapy, although it requires further investigations.

## Background

Cancer is considered as a serious concern affecting the health of all human societies. Prostate cancer (PC) is the most common malignancy in males worldwide, representing the second leading cause of mortality [1]. Various environmental factors such as lifestyle, physical activity, obesity and dietary habits, particularly high intake of saturated fats, have been related to an increased risk of PC [2]. Arachidonic acid (AA), one of the most important polyunsaturated fatty acids (PUFAs), constitutes an essential part of our diet and is found in meat, egg and dairy products [3]. This important fatty acid is required to maintain normal physiological functions, including tissue regeneration, cell membrane fluidity [4] and immune response [5]. However, excessive consumption of this fatty acid has an important role in cancer development [6] and this link has been established in numerous studies [7, 8]. For example, experiments on mouse models supported the effects of high-fat diet (HFD) in development and progression of prostate cancer [9, 10]. Moreover, it has been demonstrated that HFD results in increased tumor weight and number of intestinal polyps in mice [11, 12]. Similar effects have been reported in mouse models with breast cancer [13, 14].

Human lipoxygenases (LOXs) are a family of non-heme iron based enzymes that are involved in the metabolism of AA and other PUFAs into biologically active metabolites [15]. So far, several human LOX isoforms including 15-LOX-1, 15-LOX-2, 5-LOX and 12-LOX have been identified and categorized based on enzymatic characterization [16]. For example, 15-LOXs introduce the oxygen at carbon 15 of AA backbone. Two distinct human 15-LOXs are widely distributed in various tissues, exhibiting different substrate preferences. 15-LOX-1, one of the critical enzymes of mammalian LOX family, is physiologically overexpressed in specific cancerous tissues such as prostate [17] and renal cancer [18], while it is poorly expressed in colorectal [19, 20], lung [21], esophageal [22] and bladder cancers [23] as well as chronic myeloid leukemia [24]. Thus, 15-LOX-1 is involved in both tumor promotion and suppression depending on the type of cancer. This enzyme predominantly metabolizes linoleic acid (LA) to 13*S*-hydroperoxyoctadecadienoic acid (13*S*-HpODE), but also metabolizes AA to 12*S*- and 15*S*-hydroperoxyeicosatetraenoic acid (15*S*-HpETE) [25]. 15-LOX-2 is constitutively expressed in healthy normal prostate tissue [17] while it is down regulated in prostate [26], lung [27] and esophageal cancers [28]. In contrast to 15-LOX-1, 15-LOX-2 preferentially catalyzes AA to 15*S*-HpETEs and poorly metabolizes LA [29]. Similarly, 5-LOX and 12-LOX utilize AA to synthesize 5*S*-HpETE and 12*S*-HpETE, respectively [30, 31] and their expression is linked to PC spreading and metastasis [32]. In this context, blocking overexpressed LOX enzymes in PC could be one of the promising targets facilitating drug discovery and treatment. There are several studies reporting different compounds with significant inhibitory effects on 15-LOX-1. Coumarin-based inhibitors, such as 8-farnesyloxycoumarin [33], 5-farnesyloxycoumarin [34] and 7-farnesyloxycoumarin [35] exhibited inhibitory potency against 15-LOX-1 with half maximal inhibitory concentration (IC_50_) values of 2.64, 8.32 and 11.8 µM, respectively. Furthermore, the inhibitory activities of stylosin and its derivatives have also been shown on soybean LOX-1 [36]. These compounds exerted their anti-cancer effects in PC cells via induction of apoptosis. In addition, indole-based inhibitors, such as 14d [37], indolylpyrazoline (4b) [38] and 9c (i472) [39] are members of another group of potent 15-LOX-1 inhibitors. Sarveswaran et al*.* [40]*,* treated PC cells with MK591 as a specific chemical inhibitor of 5-LOX activity in order to induce apoptosis via down-regulation of protein kinase C-epsilon (PKCε) and disruption of c-Myc signaling [41]. Moreover, it has been shown that inhibition of 12-LOX activity markedly sensitizes PC cells to radiation both in vivo and in vitro [42, 43]. Recent studies have demonstrated that ferroptotic pathway as another type of programmed cell death can be mediated via LOXs in some contexts to eradicate cancers [44, 45].

Considering the association between lipoxygenase activity and PC development, the inhibition of these pathways provides an interesting therapeutic strategy to suppress tumorigenesis and malignant growth. It has been shown that 4-methyl-2-(4 methylpiperazinyl)pyrimido[4,5-b]benzothiazine (4-MMPB) as a heterocyclic compound, functions as an effective and specific 15-lipoxygenase inhibitor with an IC_50_ value of 18 µM [46]. Moreover, its analog, 4-propyl-2-(4-methylpiperazinyl)pyrimido[4,5-b]benzothiazine (4-PMPB), exhibited robust inhibitory potency against soybean LOX-1 with an IC_50_ value of 9 µM [47]. In this study, inhibitory potency of the other analog with ethyl moiety at position 4 of pyrimido benzothiazine (4-ethyl-2-(4-methylpiperazinyl)pyrimido[4,5-b]benzothiazine (4-EMPB)) against lipoxygenases was also investigated (Table 1). We aimed to test the anti-cancer effects of 4-PMPB and 4-EMPB on PC cells both in vitro and in vivo. Cytotoxicity and anti-cancer properties of the three compounds were evaluated on cancerous (human prostate cancer cell line; PC-3) and normal (human dermal fibroblast; HDF) cells. Moreover, the mechanism of cell death was assessed via FITC-annexin V and propidium iodide (PI) staining. Tumor inhibitory effects of the compounds were then evaluated in immunocompromised C57BL/6 mice bearing human PC-3 cells.

**Table 1:** Chemical characteristics of 4-MMPB and its analogs

Compound	Chemical structure	Molecular weight (g/mol)
4-Methyl-2-(4-methylpiperazinyl)pyrimido[4,5-b]benzothiazine (4-MMPB)		314.4
4-Propyl-2-(4-methylpiperazinyl)pyrimido[4,5 b]benzothiazine (4-PMPB)		343
4-Ethyl-2-(4-methylpiperazinyl)pyrimido[4,5-b]benzothiazine (4-EMPB)		329

## Methods

All chemical reagents and solvents were purchased from Merck (Darmstadt, Germany) and used as received without further purification. 3-[4,5-dimethylthiazol-2-yl]-2,5 diphenyltetrazolium bromide (MTT) and trypsin–EDTA were purchased from Tinab Shimi (Mashhad, Iran). Roswell Park Memorial Institute 1640 (RPMI 1640) culture medium and Dulbecco’s modified Eagle’s medium (DMEM) were bought from Gibco (Scotland, UK). Fetal bovine serum (FBS; Schwalbach, Germany), and penicillin–streptomycin were obtained from Gibco (Loughborough, UK). Soybean LOX-1 (L1; type I-B; EC 1,13,11,12) and linoleic acid (L1376) were bought from Sigma‐Aldrich (St. Louis, USA). Itraconazole and co-amoxiclav were purchased from Tehran Darou Pharmaceutical Co. and AFA Chemi, Pharmaceutical Co. (Tehran, Iran), respectively.

### Synthesis of compounds

**Fig. 1:**
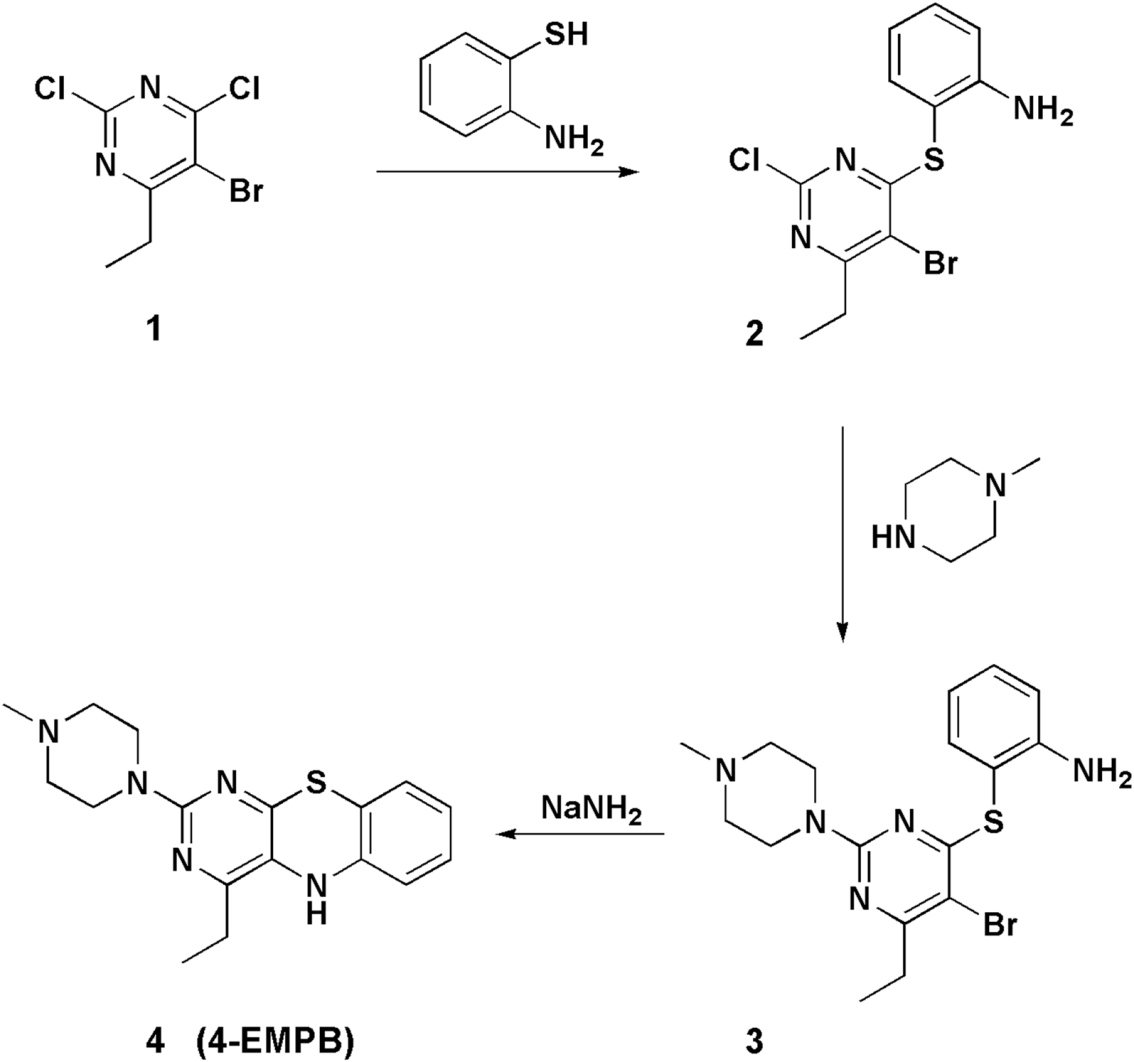
Synthetic pathway of 4-EMPB

4-MMPB and 4-PMPB were synthesized as previously described [46, 47]. The new analog 4-EMPB was similarly synthesized via the following procedure (as illustrated in Fig. 1).

### 2-[(5-Bromo-2-chloro-6-ethylpyrimidin-4-yl)sulfanyl]aniline (2)

To a solution of 5-bromo-2,4-dichloro-6-ethylpyrimidine 1 (CAS No. 1373331–48-6) (2.54 g, 10 mmol) and triethylamine (1.2 g) in chloroform (30 ml), 2-aminothiophenol (1.25 g, 10 mmol) was added dropwise with vigorous stirring over a minute. Solvent was removed and the residue was washed with warm water and then crystallized from ethanol (2.6 g, 83% yield, Melting Point: 153 °C (dec). IR: 3300, 3432 cm^−1^, ^1^H NMR (CDCl_3_): 1.27 (t, 3H, CH_3_), 2.87 (q, 2H, CH_2_), 4.30 (broad, 2H, NH_2_), 6.77–7.43 (m, 4H), ^13^C NMR (CDCl_3_): 14.1, 29.8, 114.4, 117.5, 118.5, 120.4, 128.0, 128.7, 147.3, 157.4, 161.5, 162.8).

### 4-ethyl-2-(4-methylpiperazinyl)pyrimido[4,5-b]benzothiazine (4-EMPB) (4)

A mixture of 2-[(5-bromo-2-chloro-6-ethylpyrimidin-4-yl)sulfanyl]aniline 2 (3.43 g, 10 mmol) and 4-methylpiperazin (30 mmol) in ethanol (20 ml) was heated under reflux for 5 h. Water (40 ml) was added to the solution and separated sticky liquid (intermediate 3) was dried at 80 °C for an hour and immediately utilized without purification. Sodium amide (1.2 g, 30 mmol) was added to stirred solution of the intermediate 3 in acetonitrile (30 ml), and then heated under reflux condition (3 h). The solvent was removed and a solution of 2% acetic acid (20 ml) was added to the residue and filtered. Then the residue was crystallized from benzene to give 4-ethyl-2-(4-methylpiperazinyl)pyrimido[4,5-b]benzothiazine (4-EMPB) (4) as a yellow powder (55% yield). Melting Point: 171–173 °C; IR: 3348 cm^−1^, ^1^H NMR: (CDCl_3_) 1.26 (t, 3H, CH3), 2.34 (m, 7H, CH_3_N(CH_2_)_2_), 2.86 (t, 2H, CH_2_), 3.73 (t, 4H, 2(CH_2_N)), 6.92 (dd, 2H, C7H & C8H), 7.35 (d, 1H, C6H), 8.20–8.42 (d, 2H, C9H & NH); 13C NMR: (CDCl_3_) 14.1, 23.9, 39.5, 44.1, 46.7, 56.4, 94.7, 120.5, 122.7, 124.1, 131.1, 136.7, 141, 155.6, 159.6, 167.0; Elemental analysis of C_17_H_21_N_5_S: Calculated: C, 62.36; H, 6.46; N, 21.39; S, 9.79. Found: C, 62.41; H, 6.49; N, 21.27; S, 9.72.

### Enzyme inhibitory assessment

Linoleic acid and two assay solutions (A and B) were prepared in advance. Solution A was 50 mM 3-(dimethylamino)benzoic acid (DMAB) in l00 mM phosphate buffer (pH 7.0). Solution B was a mixture of l0 mM 3-methyl-2-benzothiazolinonehydrazone hydrochloride (MBTH; 3 ml), and hemoglobin (5 mg ml^−1^, 3 ml) in 50 mM phosphate buffer at pH 5.0 (25 ml). A linoleic acid solution was prepared by mixing 5.6 mg linoleic acid with 0.5 ml methanol and then diluted with 100 mM KOH to a final volume of 5 ml (4 mM). In the assay, a solution of inhibitors in dimethyl sulfoxide (DMSO) (12.5 μl), soybean LOX-1 (4000 units ml^−1^ in 50 mM phosphate buffer pH 7.0; 25 μl), and phosphate buffer, pH 7.0 (50 mM; 435 μl) were mixed in 48-well plates, and pre incubation was carried out for 10 min at 30 °C. A control test was performed with the same volume of DMSO. After the pre incubation, linoleic acid solution (25 μl) was added to start the peroxidation reaction at 30 °C, and 10 min later, solution A (135 μl) and then solution B (65 μl) were added to start the color formation. After 3 min, 100 μl of a 2% sodium dodecyl sulphate (SDS) solution was added to terminate the reaction. The absorbance at 598 nm was compared with the control. These experiments were performed in triplicate and the data were analyzed using GraphPad Prism 6.0 (San Diego, USA).

### Preparing different solutions of compounds for cell culture

Two mg of each compound was dissolved in 100 μl hydrochloric acid (HCl 0.3 N) to make a primary stock solution. Different concentrations (1.56–50 μg ml^−1^) were then prepared by serial dilution from the primary stock solution and further diluted in the culture medium. Moreover, culture medium containing 0.25% HCl 0.3 N was used as a negative control in all experiments for a better evaluation.

### Cell culture

PC-3 cells were obtained from Pasteur Institute (Tehran, Iran) and routinely cultured in RPMI 1640 supplemented with 10% FBS. In order to investigate the toxicity of the compounds on normal cells, in vitro experiments were also performed on HDF cells. These cells were kindly provided by Academic Center for Education, Culture and Research (ACECR, Mashhad, Iran) and grown in DMEM supplemented with 10% FBS. Both cell lines were kept at 37 °C containing 5% CO_2_ in a humidified incubator.

### Cytotoxicity assay

The MTT reduction assay is a standard quantitative method to evaluate cytotoxicity. This assay measures cellular dehydrogenases, and is considered as an easy, safe and highly reproducible method. Viable cells reduce yellow tetrazolium salt to purple formazan crystals and thus the color change serves as a marker reflecting cell viability [48].

PC-3 and HDF cells were seeded in 96-well plates (NEST, China) at a density of 6000 and 8000 cells per well, respectively and incubated at 37 °C with 5% CO_2_. Next day, the media were changed and both cell lines were treated with various concentrations (1.56–50 μg ml^−1^) of the compounds and cisplatin. Experiments were carried out in triplicate for 4-PMPB, 4-EMPB and 4-MMPB as well as cisplatin as a positive control. After 24, 48 and 72 h of treatments, 20 μl MTT solution (5 mg ml^−1^ in PBS; phosphate buffered saline) was added to each well, and cells were incubated in the dark for 3–4 h. The media were then removed and purple crystals were dissolved in 160 μl DMSO. The absorbance in each well was measured at 540 nm as reference wavelength in an enzyme-linked immunosorbent assay (ELISA) reader (Awareness Technology, USA) and relative optical density (OD) values were recorded. The percentage of viable cells was calculated using the following equation [49]:

$${\text{Cell viability }}\left( \% \right) = {1}00 \times \left[ {\left( {{\text{OD}}_{{{\text{mean}}}} {\text{of treated cells}} - {\text{OD}}_{{{\text{mean}}}} {\text{blank}}} \right)/\left( {{\text{OD}}_{{{\text{mean}}}} {\text{of negative control cells }}\left( {{\text{HCl}}0.{\text{3 N}}} \right) - {\text{OD}}_{{{\text{mean}}}} {\text{blank}}} \right)} \right].$$

### Flow cytometric analysis

To determine the mechanism of cell death induced by 4-MMPB analogs, flow cytometry was employed using the Annexin V-FITC kit with PI (BioLegend, San Diego, USA) according to the manufacturer’s instructions. We performed co-treatment experiments with the compounds and a ferroptosis inhibitor (IC_50_ values of 4-PMPB + , 4-EMPB + , and 4-MMPB + deferoxamine (80 µM)) on PC-3 cells, to distinguish between ferroptosis and apoptosis mechanisms [50]. In order to evaluate the apoptosis mechanism, cells were treated with the ½ × IC_50_ and IC_50_ values of 4-PMPB, 4-EMPB, 4-MMPB, and cisplatin for 24 h. Then the cells were collected by trypsinization (0.25% trypsin-1 mM EDTA), washed twice with PBS, followed by centrifugation, and resuspended in a binding buffer. Samples were stained with Annexin V-FITC/ PI followed by 15 min incubation at room temperature. Finally, cells were subjected to flow cytometry (BD Accuri C6, San Jose, USA) and the data were analyzed by FlowJo 7.6.1 software (Ashland, USA). Moreover, PC-3 cells treated with 0.3 N HCl were used as a control to eliminate the effects of solvent.

### Anti-cancer evaluation of compounds on a PC-3 xenograft tumor model

The animal experiments were approved and conducted in accordance with Animal Ethics Committee of Ferdowsi University of Mashhad (FUM) (IR.UM.REC.1399.001). C57BL/6 mice were inbred in an animal house at FUM and maintained under pathogen-free conditions. Immunosuppression of male C57BL/6 mice (4–6 weeks old) was carried out according to the protocol described by Jivrajani et al. [51]. However, in this study, for immunosuppression ketoconazole and ampoxin drugs were replaced with itraconazole and co-amoxiclav. After completion of this procedure, the total white blood cell (WBC) and lymphocyte counts were evaluated to confirm the immunosuppression protocol.

The PC-3 tumor model was generated by subcutaneous injection of 3 × 10^6^ cells in 100 μl RPMI 1640 into the right flank of the immunocompromised mice. When the tumor volume reached 100–200 mm^3^, the animals were randomly divided into 9 groups (n = 8 per group) and treated with: 1) PBS as a negative control; 2) HCl (0.3 N) as solvent control; 3) 4-PMPB (10 mg kg^−1^); 4) 4-PMPB (50 mg kg^−1^); 5) 4-EMPB (10 mg kg^−1^); 6) 4-EMPB (50 mg kg^−1^); 7) 4-MMPB (10 mg kg^−1^); 8) 4-MMPB (50 mg kg^−1^) and 9) cisplatin as a positive control (2 mg kg^−1^). The changes in tumor size and body weight were monitored every other day. The tumor volumes were calculated using the following formula [52]:

$${\text{Tumor volume }}\left( {{\text{mm}}^{{3}} } \right) = \left( {\text{Tumor length}} \right) \times \left( {\text{Tumor width}} \right)^{{2}} \times 0.{5}$$

To validate the anti-tumor activity in different experimental groups, mice were sacrificed 15 days post treatment. Meanwhile, blood samples were collected and histological analyses were also performed to investigate the possible side effects. Hematological analyses were performed in an automated hematology analyzer (Sysmex XP-300; Lincolnshire, USA). The tissue slices were stained with hematoxylin and eosin (H&E) and observed by an optical microscope (Olympus IX70; Tokyo, Japan).

### Statistical analysis

All statistical analyses were carried out using GraphPad Prism software 6.0 (San Diego, USA). First the Kolmogorov–Smirnov test was performed to evaluate the normal distribution of the data. Then, differences between groups were assessed with Student’s *t*-test (group pairs) or one-way ANOVA (multiple groups) followed by Tukey multiple comparison tests. Experiments were performed in triplicate and data are presented as mean ± standard error or standard deviation as required. *p*-values < 0.05 were considered as significant.

## Results

### Enzyme activity assay

**Fig. 2:**
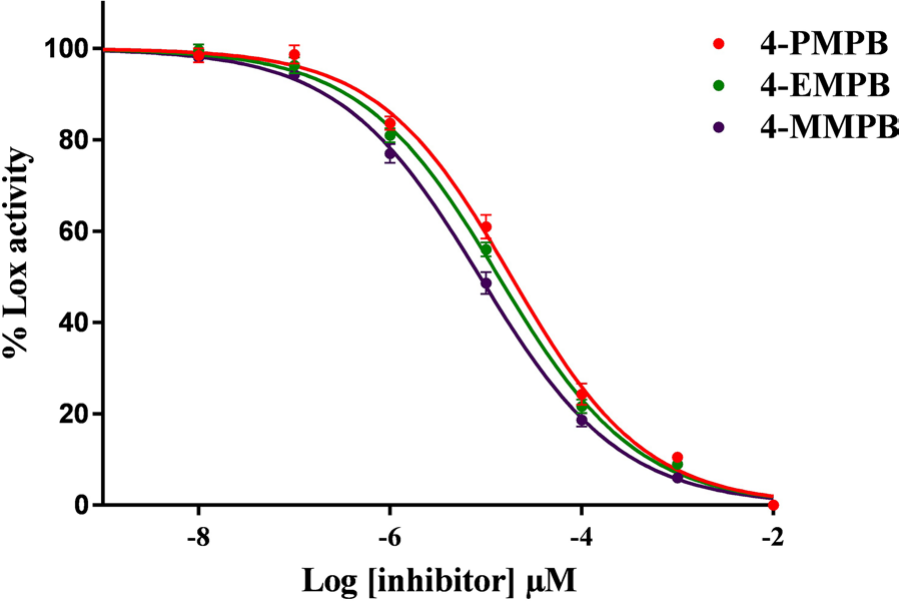
Dose–response curves for soybean LOX-1 inhibition by 4-MMPB and its analogs. Data are expressed as mean ± SEM, n = 3

Lipoxygenase inhibitory activity of 4-MMPB and its analogs was assessed on soybean LOX-1 and the inhibitory potencies (IC_50_ values) of the mentioned compounds were calculated from their sigmoidal dose–response curves (Fig. 2). As expected, 4-EMPB decreased lipoxygenase activity by IC_50_ value of 14.3 μM, which falls between inhibitory potencies of 4-PMPB (IC_50_ = 8.6 μM) and 4-MMPB (IC_50_ = 17.1 μM).

### In vitro cell viability assay

**Fig. 3:**
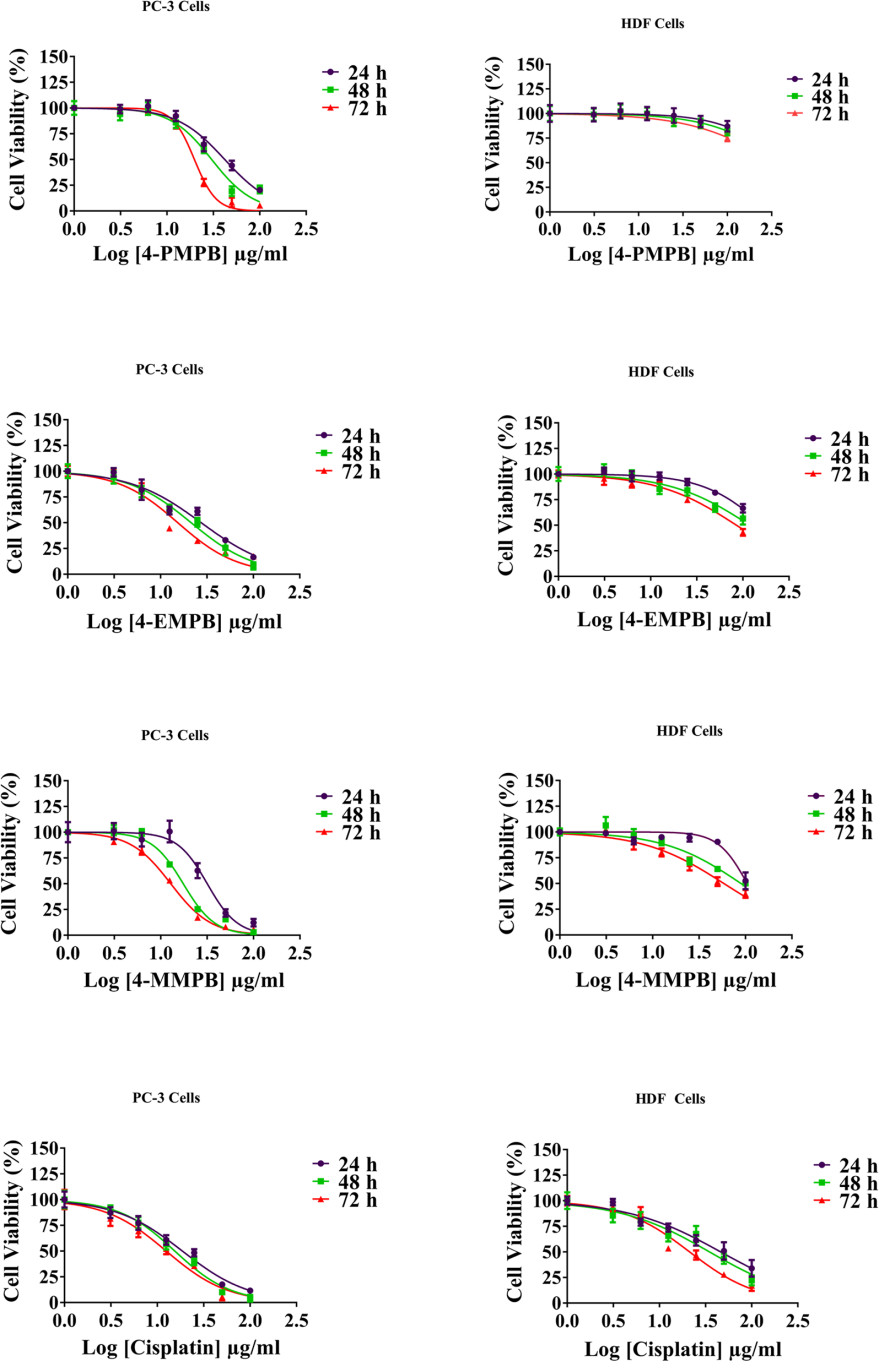
Dose–response curves of different compounds on PC-3 and HDF cells as evaluated by MTT assay. Cells were treated with 4-PMPB, 4-EMPB, 4-MMPB and cisplatin for 24, 48, and 72 h. Data are expressed as mean ± SEM, n = 3

In present study, we first evaluated in vitro cytotoxicity and anti-cancer properties of 4-PMPB and 4-EMPB on PC-3 and HDF cells, and the results were compared with 4-MMPB as a potent 15-lipoxygenase inhibitor and cisplatin as a well-known chemotherapeutic drug. After exposing both cell lines to different concentrations of compounds, cell viability was determined by MTT assay and IC_50_ values were calculated. The IC_50_ values and dose–response curves are presented in Table 2 and Fig. 3, respectively. Dose–response curves indicated that 4-PMPB and 4-EMPB have similar anti-cancer effects on PC-3 cells as compared with 4-MMPB. As shown in Table 2, 4-PMPB, showed no obvious cytotoxicity on normal cells, while, 4-EMPB and 4-MMPB exhibited a weak cytotoxicity on HDF cells. Furthermore, cisplatin showed prominent cytotoxic effects on both prostate cancer and normal cells.

**Table 2:** Anti-proliferative activity of tested compounds on PC-3 and HDF cells during 24, 48, and 72 h of treatments

Compounds	IC_50_ (µM) ± SD (PC-3)			IC_50_ (µM) ± SD (HDF)		
	24 h	48 h	72 h	24 h	48 h	72 h
4-PMPB	122.303 ± 2.618	88.45 ± 2.312	57.58 ± 2.123	1641.10 ± 0.075	1443.73 ± 0.055	1028.86 ± 0.139
4-EMPB	107.44 ± 6.17	74.95 ± 3.39	48.99 ± 6.76	545.55 ± 1.99	361.70 ± 2.29	258.87 ± 1.86
4-MMPB	100.76 ± 2.412	55.55 ± 1.980	40.79 ± 1.795	331.42 ± 3.332	284.09 ± 2.975	185.61 ± 2.805
Cisplatin	62.29 ± 1.741	51.53 ± 1.425	41.63 ± 1.108	153.42 ± 2.89	119.49 ± 2.565	71.09 ± 3.337

### Evaluating the mechanism of cell death

**Fig. 4:**
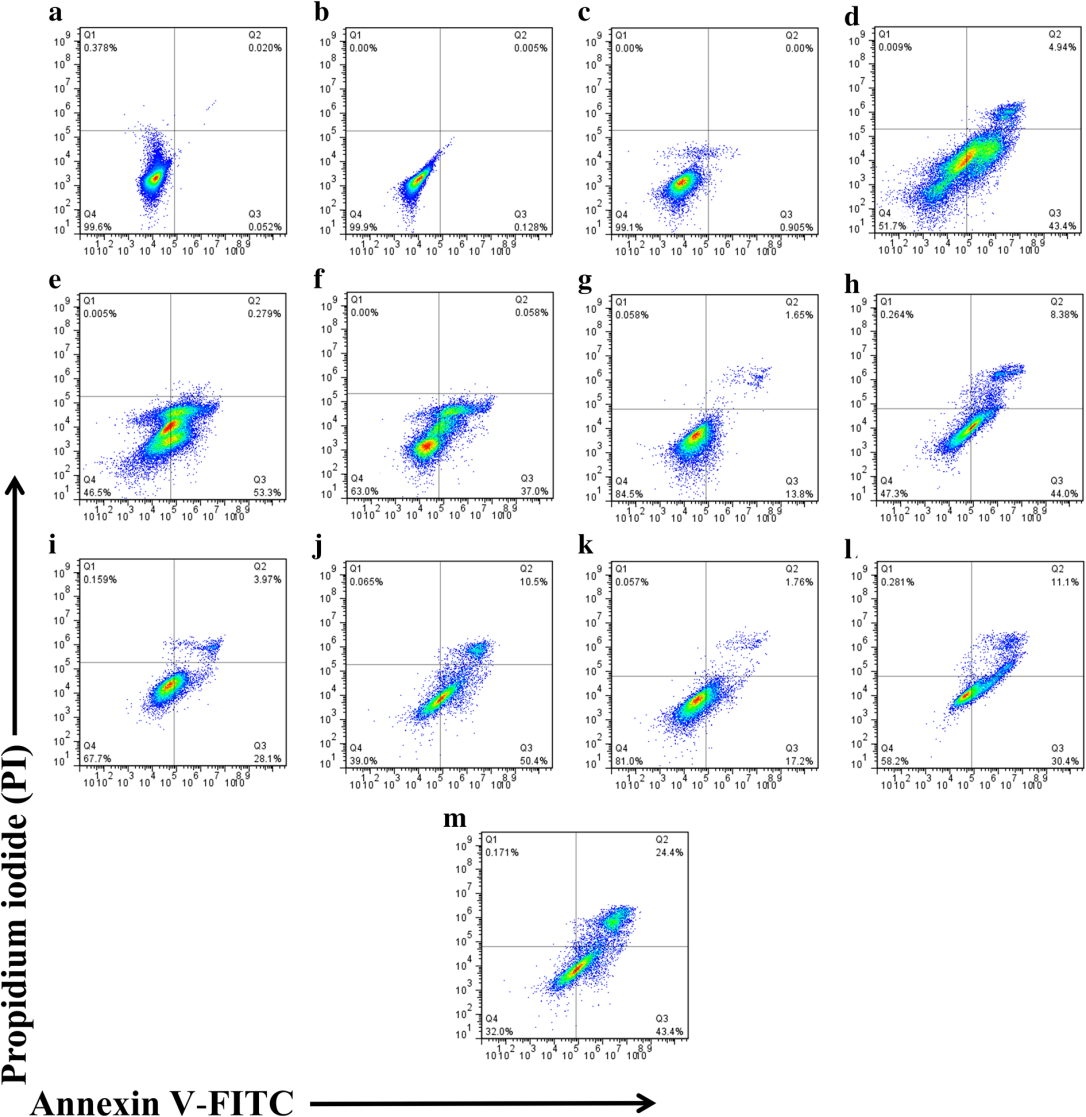
Flow cytometric analysis of cell death induced in PC-3 cells treated with deferoxamine, $\frac{1}{2}$× IC_50_ and IC_50_ values of each compound after 24 h. **a** Untreated, **b** 0.3 N HCl, **c** deferoxamine (80 µM), **d** 4-PMPB + deferoxamine (122.303 + 80 µM), **e** 4-EMPB + deferoxamine (107.44 + 80 µM), **f** 4-MMPB + deferoxamine (100.76 + 80 µM), **g** 4-PMPB (61.15 µM), **h** 4-PMPB (122.303 µM), **i** 4-EMPB (53.72 µM), **j** 4-EMPB (107.44 µM), **k** 4-MMPB (50.38 µM), **l** 4-MMPB (100.76 µM) and **m** cisplatin

To evaluate the mechanism of cell death, PC-3 cells co-treated with 4-PMPB, 4-EMPB, or 4-MMPB in combination with deferoxamine, a ferroptosis inhibitor, were assessed using Annexin V-FITC/ PI staining and compared with those treated with the compounds only. The results revealed that the population in Q2 (Annexin V positive/PI positive) was markedly decreased after co-treatment with deferoxamine as compared to those evaluated in the absence of this ferroptosis inhibitor. These observations suggest that 4-MMPB and its two analogs have probably induced both apoptosis and ferroptosis in PC-3 cells (Fig. 4). Flow cytometry–based quantification showed the percentage of early and late apoptotic/ ferroptotic cells (Q3_+_Q2) increased from 0.133% in control to 52.38%, 60.9%, 41.5% and 67.8% in 4-PMPB-, 4-EMPB-, 4-MMPB-, and cisplatin- treated PC-3 cells, respectively, when treated with IC_50_ values of the compounds.

### In vivo anti-tumor effects of the compounds

**Fig. 5:**
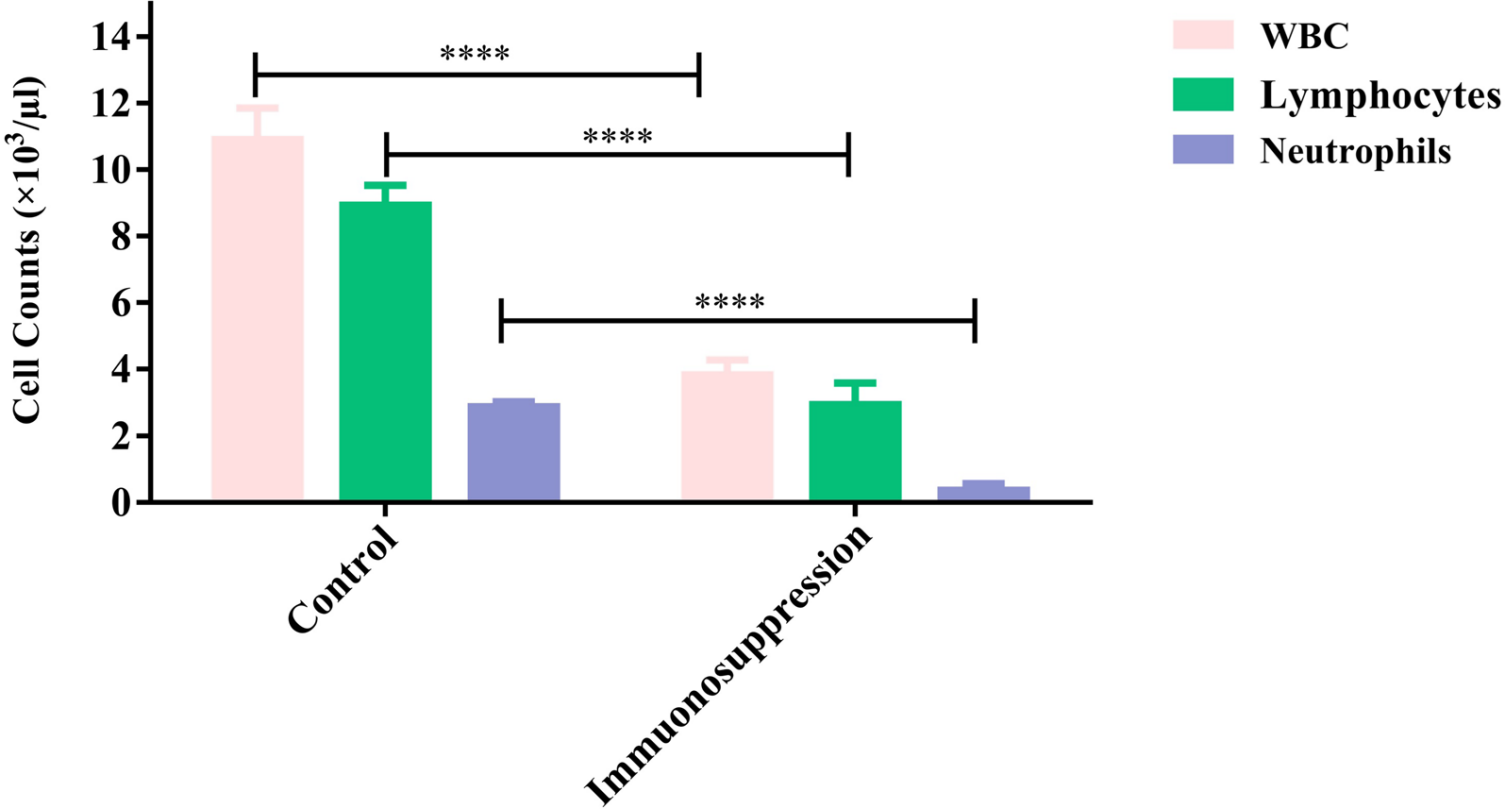
Effects of immunosuppression protocol on white blood cell (WBC), lymphocyte and neutrophil counts. Significant differences were observed between control and immunosuppression groups. Data are expressed as mean ± SD, n = 3, **** *p* < 0.0001

**Fig. 6:**
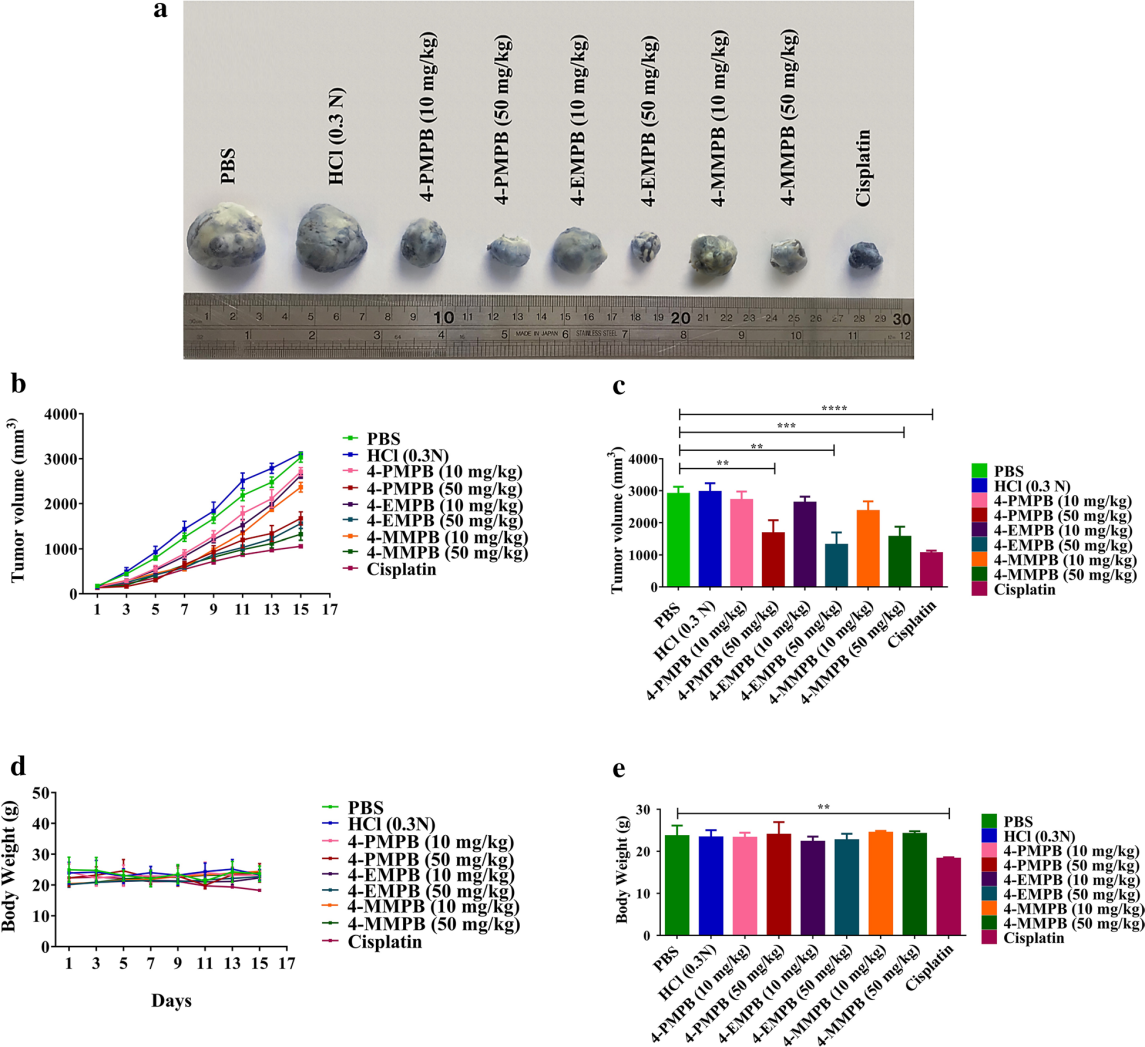
In vivo therapeutic efficacy of 4-PMPB, 4-EMPB and 4-MMPB on immunocompromised C57BL/6 mice bearing PC-3 tumors. **a** Representative image of tumors at day 15 after various treatments. **b** Tumor growth curves indicated, cisplatin and high doses of 4-PMPB, 4-EMPB and 4-MMPB had remarkable anti-tumor effects during the 15 days of treatments. **c** At the end of the experiment, tumor volumes were measured and significant differences were observed between the PBS and HCl controls and those treated with 50 mg kg^-1^ of the compounds as well as cisplatin groups. **d** The body weights of different groups were continuously evaluated during the 15 days follow up. **e** There was a significant difference in body weight loss between cisplatin and other treatment groups on day 15. Data are expressed as mean ± SD, n = 8. ** *p* < 0.01, *** *p* < 0.001 and **** *p* < 0.0001

After administration of immunosuppression protocol, blood samples were collected and their parameters were compared with normal C57BL/6 mice. Significant reduction in WBC, lymphocyte, and neutrophil counts (*p* < 0.0001) was confirmed in immunocompromised mice (Fig. 5). On day 11 after immunosuppression, PC-3 cells were injected subcutaneously. Immunocompromised mice bearing PC-3 tumors were then treated with PBS, 0.3 N HCl, 4-PMPB (10 and 50 mg kg^−1^), 4-EMPB (10 and 50 mg kg^−1^), 4-MMPB (10 and 50 mg kg^−1^), and cisplatin, and then their body weights and tumor volumes were measured every other day. As shown in Fig. 6a–c, tumor growth was effectively inhibited after treatment with high doses of 4-PMPB, 4-EMPB, and 4-MMPB. In contrast, tumors in control groups receiving PBS and 0.3 N HCl grew markedly during the 15 days follow up period. Furthermore, the positive control group, receiving cisplatin, exhibited a significant decrease in tumor growth as compared with the controls and other experimental groups with lower doses. The results revealed that, there was no significant difference between tested compounds (4-PMPB, 4-EMPB) and 4-MMPB in tumor inhibition.

### In vivo side effects evaluation

The side effects of the compounds were evaluated by measuring the body weights, hematological analyses, and H & E staining of different organs. Body weights of various groups were compared and as shown in Fig. 6d and e, the cisplatin treated group demonstrated the lowest body weights, which were significantly lower than other experimental groups (*p* < 0.01). However, no significant changes in body weights were observed between 4-PMPB- (10 and 50 mg kg^−1^), 4-EMPB- (10 and 50 mg kg^−1^) and 4-MMPB- (10 and 50 mg kg^−1^) treated groups.

**Fig. 7:**
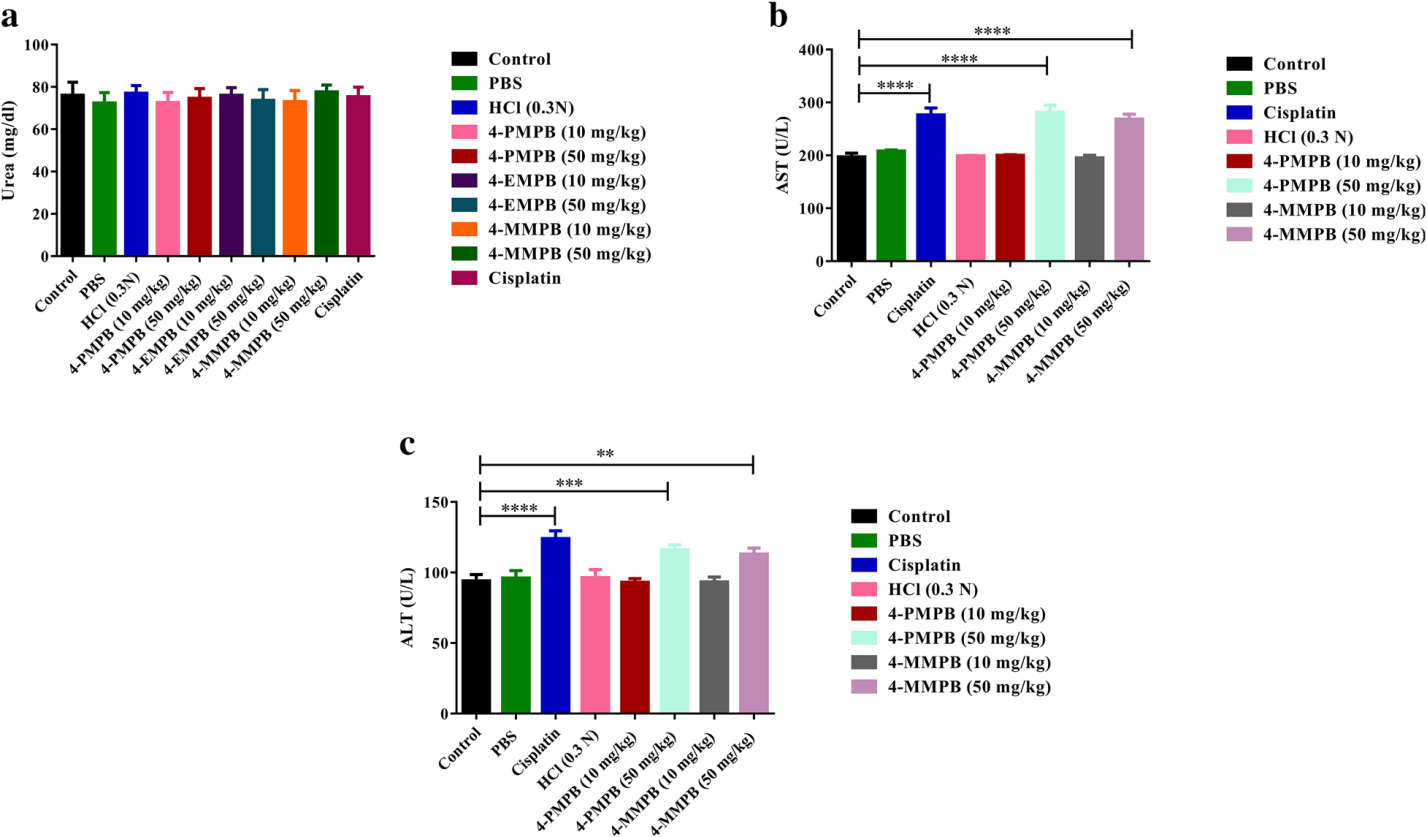
Effects of different treatments on serum biochemical parameters including **a** urea, **b** aspartate transaminase (AST) and **c** alanine aminotransferase (ALT). Data are expressed as mean ± SD, n = 3, ** *p* < 0.01, *** *p* < 0.001 and **** *p* < 0.0001

**Fig. 8:**
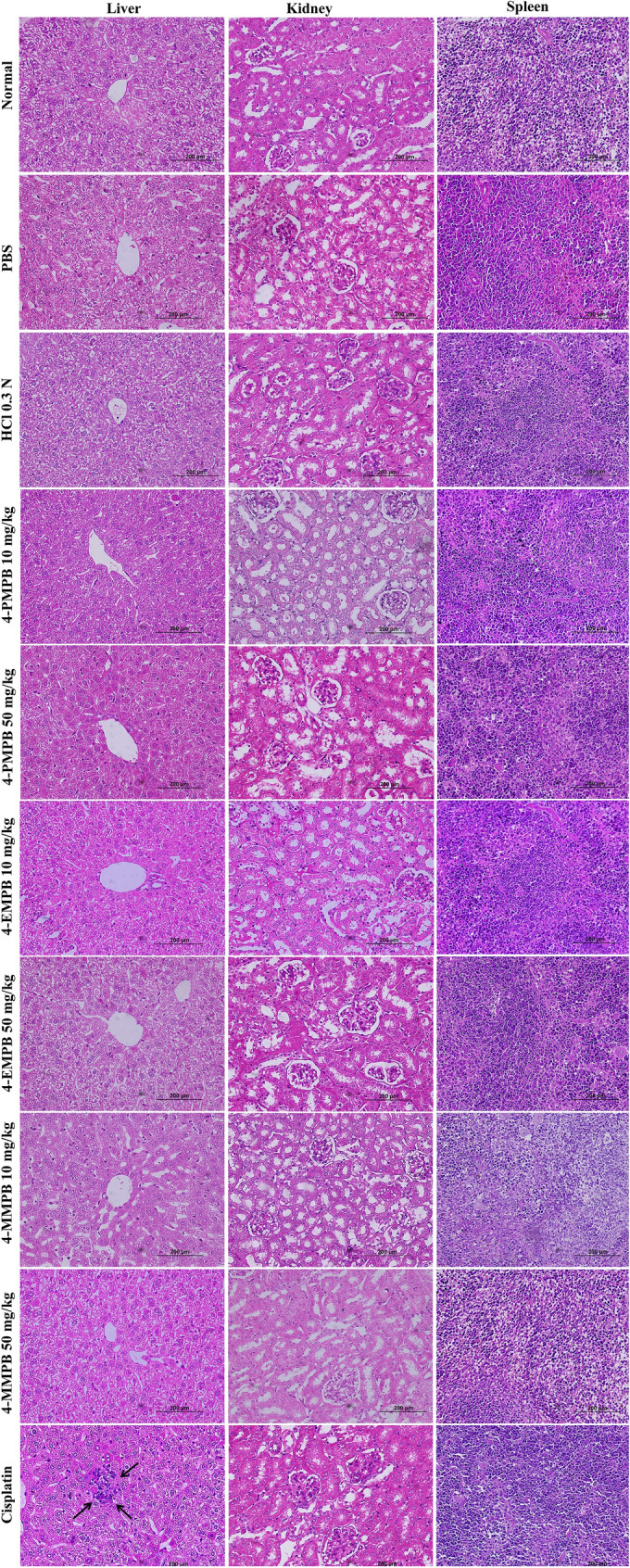
Hematoxylin and eosin (H&E)-stained images of major organs (liver, kidney, and spleen) collected on day 15 after treatments. Mild and local accumulation of inflammatory cells was noticed in cisplatin treated group (black arrows). Scale bar: 200 μm

**Fig. 9:**
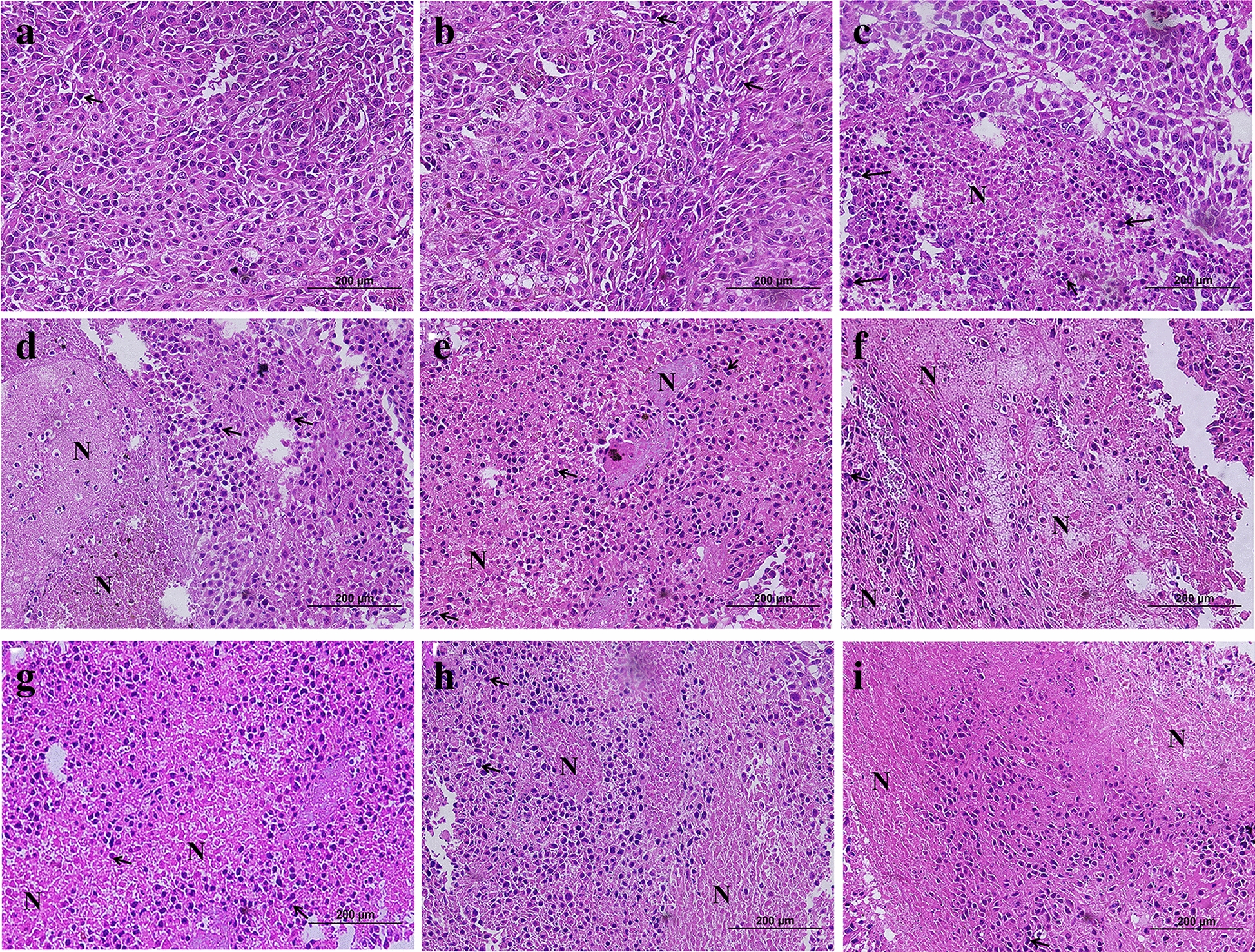
Hematoxylin and eosin (H & E)-stained images of tumor tissues upon different experimental conditions: **a** PBS, **b** 0.3 N HCl, **c** 4-PMPB (10 mg kg^−1^), **d** 4-PMPB (50 mg kg^−1^), **e** 4-EMPB (10 mg kg^−1^), **f** 4-EMPB (50 mg kg^−1^), **g** 4-MMPB (10 mg kg^−1^), **h** 4-MMPB (50 mg kg^−1^) and **i** cisplatin. Black arrows and “N” represent mitotic figures and necrotic areas, respectively within the tumor mass. Scale bar: 200 μm

To determine possible side effects of various treatments in vivo, biochemical parameters such as urea, aspartate transaminase (AST) and alanine aminotransferase (ALT) were evaluated in different experimental groups (Fig. 7). Urea levels did not change in all treatment groups as compared with the immunosuppressed group. Mice treated with 4-PMPB, 4-EMPB and 4-MMPB at concentrations of 50 mg kg^−1^ had significantly elevated AST and ALT levels compared with immunosuppressed control group. Similarly, the group treated with cisplatin had significantly increased serum liver enzymes compared to control group (*p* < 0.0001). Histological analysis showed no obvious tissue damages in major organs including liver, kidney, and spleen, following 4-PMPB, 4-EMPB, and 4-MMPB treatments. However, mild and local accumulation of inflammatory cells was noticed in cisplatin treated group (Fig. 8). Meanwhile, the tumor tissue treated with cisplatin and high doses of compounds exhibited a higher degree of cell death than the other experimental groups (Fig. 9). Apoptosis of tumor cells with nuclear and cytoplasmic condensation and also tumor necrosis were visualized in treated groups. However, further immunohistochemical analyses are required to confirm the exact type of cell death in tumor tissues.

## Discussion

In recent years, cancer incidence rate has increased significantly especially in developing countries and discovering more effective treatments has emerged as a major concern. Chemotherapy is one of the conventional treatment modalities, which can increase patient’s survival. However, therapeutic index can be decreased due to serious limitations of chemotherapy including drug resistance, poor specificity, and undesirable, long term side effects [53, 54]. Discovering cell signaling molecules which can trigger an apoptotic pathway in cancerous cells is crucial to improve therapeutic outcomes. Lipoxygenases are considered as a family of important signaling mediators that their overexpression is associated with tumor initiation and progression of prostate cancer [55]. Development of effective lipoxygenase inhibitors may successfully diminish cell proliferation in the tumor mass. An example of established 15-lipoxygenase inhibitors is 4-MMPB [46], which has been well documented in recent studies on PC-3 cells [33–36]. In this study, two 4-MMPB analogs, 4-PMPB and 4-EMPB, with inhibitory effects on lipoxygenase activity [46, 47] were employed and 4-PMPB indicated the most selective anti-proliferative effects as compared with the other two. The significantly lower cytotoxicity of these compounds on HDF cells can be attributed to the low expression of lipoxygenases in these normal cells [56, 57]. Considering the lower levels of cell death population after co-treatment with 4-PMPB, 4-EMPB or 4-MMPB compounds and deferoxamine, compared with those treated with the compounds only, it is proposed that cell death is triggered through both apoptosis and ferroptosis pathways. It has been shown that *ALOX15* expression is regulated by a mutant form of *P53* and results in promoting PC [58]. Moreover, 13*S*-HODE, the major metabolite of 15-LOX-1 can interact with anti-apoptotic mediators such as B-cell lymphoma 2 (BCL2) and insulin-like growth factor receptor-1 (IGF-1R) which leads to inhibition of apoptotic signaling pathways in PC [59–61]. Additionally, recent studies demonstrated that 15-LOX-1 is one of the fundamental regulators in ferroptosis pathway [44, 62], and is involved in PUFA oxidation and pro‐ferroptotic lipid peroxide generation [63]. In order to better evaluate the efficacy of anti-cancer agents, treatment strategy should also be assessed in animal experimental models [64, 65]. Nude and immunocompromised mice models are commonly used as hosts for human cancer cell lines. However, nude mice bear several drawbacks due to high cost, low tumorigenesis rate, high sensitivity, difficult maintenance and high mortality rate [66]; therefore we used immunocompromised mice bearing human PC as a preferred model. There are a number of immunosuppressive medications such as glucocorticoids, azathioprine, cyclophosphamide, methotrexate, cyclosporine, ketoconazole and tacrolimus which are used alone or in combination to prevent human tumor xenografts rejection in murine models [64]. Cunha et al*.* [67] reported xenotransplantation of human glioblastoma cells in rats by cyclosporine treatment (5 mg per kg per day) until the end of experiments. Similarly, Du et al*.* [68] reported use of cyclosporine treatment (10 mg per kg per day) for development of human hepatocarcinoma cells xenograft in a C57BL/6 mouse model. Moreover, Rose et al. [69] reported that xenologous skin transplantation in an immunosuppressed sheep model was made possible by using cyclosporine (2–3 mg kg^−1^) in combination with ketoconazole (10 mg kg^−1^) after 18 days. In present study, human PC xenograft was developed according to the Jivrajani’s protocol in C57BL/6 mice [51]. Blood count analysis after administration of immunosuppression protocol, and tumor growth volume in control groups confirmed successful immunosuppression and xenotransplantation in mouse model. Moreover, in vivo results demonstrated that while cisplatin treatment led to a significant decrease in tumor size, it suffered from side effects including a significant decrease in body weight. Furthermore, the highest dose of 4-PMPB and 4-EMPB (50 mgkg^−1^) possessed similar anti-cancer effects as compared with 4-MMPB, leading to delayed tumor growth without noticeable side effects in weight loss and histological assessments.

## Conclusion

Targeted therapeutic strategies such as lipoxygenase inhibition in PC can reduce tumor growth and help in improving clinical outcomes. Data generated from our study showed that, use of 4-PMPB and 4-EMPB can be considered as a treatment of choice to trigger both apoptosis and ferroptosis pathways and inhibit PC cell survival in vitro and in vivo probably via specific targeting of lipoxygenases. For this purpose, different approaches can be recommended, and use of nanotechnology-based drug delivery systems (DDSs) is one of the most attractive strategies. In this regard, mentioned compound inhibitors of lipoxygenases can be encapsulated into specific nanocarriers such as liposomes, which would enable us to use them in clinical trials. Moreover, surface of nanocarriers can be decorated with specific ligands referred as active targeting, to reduce possible side effects on normal tissues and facilitate their availability to tumor microenvironment.

## Abbreviations

µM: Micromolar
4-EMPB: 4-Ethyl-2-(4-methylpiperazinyl)pyrimido[4,5-b]benzothiazine
13-HpODE: 13-Hydroperoxyoctadecadienoic acid
13-HODE: 13-Hydroxyoctadecadienoic acid
15-HpETE: Hydroperoxyeicosatetraenoic acid
15-HETE: Hydroxyeicosatetraenoic acid
15-LOX-1: 15-Lipoxygenase-1
15-LOX-2: 15-Lipoxygenase-2
4-MMPB: 4-Methyl-2-(4-methylpiperazinyl)pyrimido[4,5-b]benzothiazine
4-PMPB: 4-Propyl-2-(4-methylpiperazinyl)pyrimido[4,5-b]benzothiazine
AA: Arachidonic acid
DMEM: Dulbecco’s modified Eagle’s medium
DMSO: Dimethyl sulfoxide
FBS: Fetal bovine serum
H&E: Hematoxylin & Eosin
HCl: Hydrochloric acid
HDF: Human dermal fibroblast
IC_50_: Half maximal inhibitory concentration
IGF-1: Insulin-like growth factor-1
MTT: 3-(4,5-Dimethylthiazol-2-yl)-2,5-diphenyltetrazolium bromide
SD: Standard deviation
